# Supplementary material for: Single-cell analysis of bronchoalveolar cells in inflammatory and fibrotic post-COVID lung disease
Source: Front Immunol. 2024 May 17;15:1372658. doi: 10.3389/fimmu.2024.1372658 (PMC11140060; doi:10.3389/fimmu.2024.1372658)
Supplement: Supplementary file 1 [file DataSheet_1.pdf]

SUPPLEMENTARY MATERIAL

Single-cell analysis of bronchoalveolar cells in inflammatory and fibrotic post-COVID lung disease

Puja Mehta†, Blanca Sanz-Magallón Duque de Estrada†*, Emma K Denneny†, Kane Foster, Carolin T Turner, Andreas Mayer, Martina Milighetti, Manuela Platé, Kaylee B Worlock, Masahiro Yoshida, Jeremy S Brown, Marko Z Nikolić, Benjamin M Chain, Mahdad Noursadeghi, Rachel C Chambers††, Joanna C Porter††, Gillian S Tomlinson††*

†These authors share first authorship.

††These authors share senior authorship.

*Corresponding authors.

SUPPLEMENTARY TABLES

Supplementary Table 1. Clinical and demographic information.

Subject ID	CT	Age range	Sex	Ethnicity	BMI	Smoking status	Steroid	Immune	Respiratory support	BAL‖‖ (days post acute-COVID)
1	Inf*	61-65	Female	Asian	23.9	Never	No	No	I&V**	97
3	Inf	46-50	Female	Asian	31.8	Never	MP‡	Tocilizumab	I&V, ECMO††	159
4	Fib†	51-55	Male	Asian	27.3	Ex	Dex§	No	HFNO‡‡	336
5	Fib	61-65	Male	White	23.2	Ex	Dex	No	I&V	314
6	Fib	56-60	Male	White	30.6	Never	Dex	No	I&V	351
7	Fib	61-65	Male	Asian	27.4	Never	MP, Pred‖	No	I&V	316
8	Fib	56-60	Female	White	22.6	Ex	Pred	Tocilizumab	I&V	272
9	Inf	41-45	Male	White	37.3	Never	No	No	Nil	111
12	Inf	66-70	Female	White	46.5	Never	Dex	No	CPAP§§	149
13	Inf	61-65	Male	White	28.7	Ex	Dex	No	HFNO	116

Clinical and demographic data are provided for all study subjects. *Inf = Inflammatory, †Fib = fibrotic, ‡MP = methylprednisolone, §Dex = dexamethasone, ‖Pred = prednisolone, **I&V = intubation and ventilation, ††ECMO = extracorporeal membrane oxygenation, ‡‡HFNO = high flow nasal oxygen, §§CPAP = continuous positive airway pressure, ‖‖BAL = bronchoalveolar lavage.

Supplementary Table 2. Cell type-specific differentially expressed genes identified in pseudobulk data.

**Full_dataset_up_inf**			
logFC	FDR	cluster	gene
2.010314	0.025086	Dendritic	*RHOB*
2.918473	4.39E-06	Prolif	*SKAP1*
2.241937	0.001254	Prolif	*CD3D*
1.637594	0.002353	Prolif	*RHOB*
1.944616	0.002504	Prolif	*CLEC2D*
1.961607	0.002504	Prolif	*CD3G*
2.293015	0.002504	Prolif	*FOS*
1.601146	0.002504	Prolif	*JUNB*
2.097024	0.005734	Prolif	*PHLDA1*
1.478716	0.013705	Prolif	*ATP2B1-AS1*
2.55377	0.013705	Prolif	*IFITM1*
1.679621	0.032128	Prolif	*PTPN7*
1.317954	0.046678	Prolif	*ICAM3*
**Full_dataset_up_fib**			
logFC	FDR	cluster	gene
1.654557	0.002084	Prolif	*SAP30*
**Tcell_up_inf**			
logFC	FDR	cluster	gene
**Tcell_up_fib**			
logFC	FDR	cluster	gene
**Myeloid_up_inf**			
logFC	FDR	cluster	gene
2.040356	0.010432	*FCN1*-Mono	*GADD45B*
2.095153	0.026693	*FCN1*-Mono	*RGS2*
1.453707	0.046217	*FCN1*-Mono	*LGMN*
2.154559	9.43E-05	IFN stim AM	*FOS*
1.651666	0.001844	IFN stim AM	*JUNB*
1.710627	0.002371	IFN stim AM	*LGMN*
1.698431	0.002683	IFN stim AM	*DUSP1*
1.309848	0.020696	IFN stim AM	*RHOB*
1.869253	4.90E-05	MT-AM	*DUSP1*
1.845162	0.000164	MT-AM	*FOS*
1.563411	0.000164	MT-AM	*JUNB*
1.236468	0.011581	MT-AM	*RHOB*
1.582065	0.04207	MT-AM	*LGMN*
1.918576	0.036913	Prolif AM	*RHOB*
**Myeloid_up_fib**			
logFC	FDR	cluster	gene
1.695279	0.048914	*FCN1*-Mono	*ZFPM1*

1.616057	0.002683	IFN stim AM	*RRAS*
1.33114	0.01932	IFN stim AM	*CA2*
1.765763	0.01323	MT-AM	*UQCRHL*
1.682482	0.020917	MT-AM	*RETN*
1.416307	0.024438	MT-AM	*MT1M*
1.489704	0.042579	MT-AM	*CCL23*

The results of differential expression analysis using a negative binomial generalized linear model with quasi-likelihood F test (GLM-QLF) performed on data aggregated to cell type pseudobulk level for each donor for the full dataset, T cells only and myeloid cells only. A false discovery rate (FDR) <0.05 was considered significant. “Up_inf” denotes genes expressed at significantly higher level in inflammatory PCLD and “up_fib” denotes genes expressed at significantly higher level in fibrotic PCLD. logFC = log2 fold change comparing inflammatory and fibrotic PCLD, cluster indicates cell type. Empty cells indicate that no genes were identified as differentially expressed between the two radiological groups in any T cell subset.

Supplementary Table 3. Shared CDR3 sequences.

Sample	CT	CDR3	Chain	Frequency
9	Inflammatory	CAVNTNAGKSTF	Alpha	1
13	Inflammatory	CAVNTNAGKSTF	Alpha	2
4	Fibrotic	CAVRDSNYQLIW	Alpha	1
6	Fibrotic	CAVRDSNYQLIW	Alpha	1
7	Fibrotic	CAVRDSNYQLIW	Alpha	1
5	Fibrotic	CAVRPRSGNTPLVF	Alpha	1
7	Fibrotic	CAVRPRSGNTPLVF	Alpha	1

CDR3 amino acid sequences found in more than one subject are listed, along with the frequency at which they were detected.

Supplementary Table 4. Virus-reactive CDR3 sequences in clusters composed of one PCLD phenotype.

Sample	CT	Cluster	CDR3 sequence	Chain	Virus
7	Fib*	82	CAVNTGFQKLVF	Alpha	SARS-CoV-2‡
13	Inf†	77	CAVGAGTNAGKSTF	Alpha	CMV§

List of virus-reactive CDR3 amino acid sequences from VDJdb detected in clusters composed uniquely of one PCLD phenotype. *Fib = Fibrotic, †Inf = inflammatory, ‡SARS-CoV-2 = severe acute respiratory syndrome coronavirus 2, §CMV = cytomegalovirus.

Supplementary Figure 1

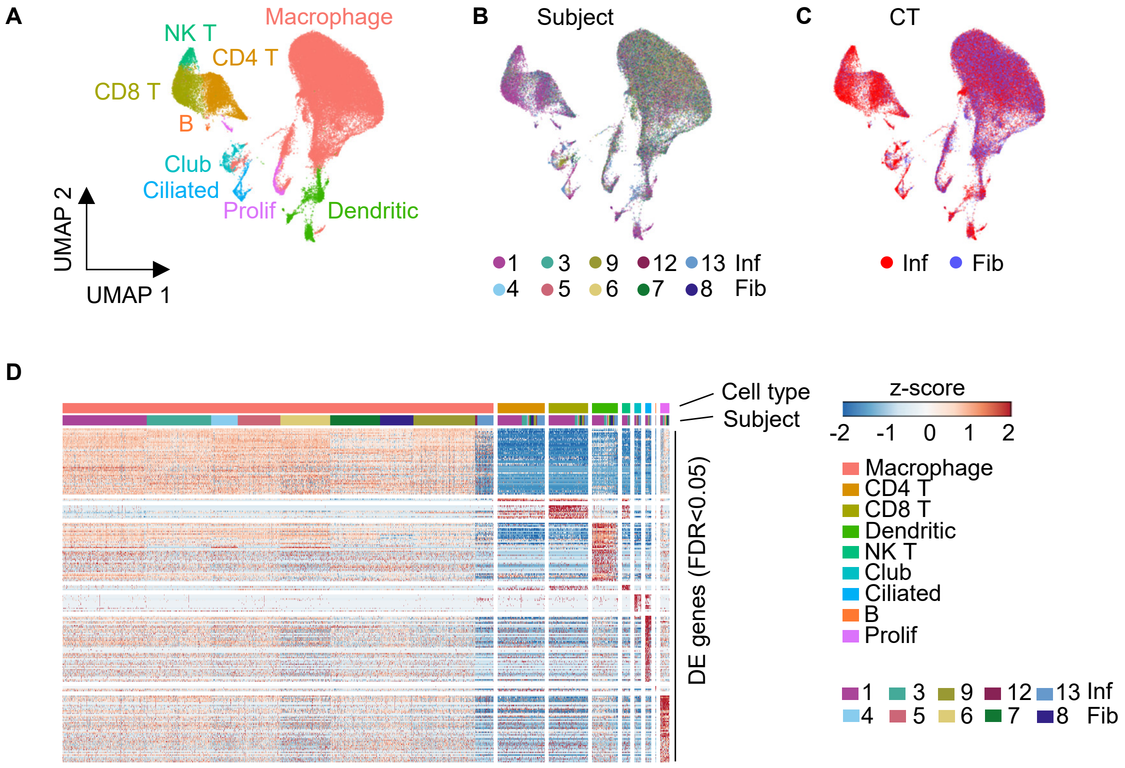

Cellular composition of post-COVID-19 lung disease (PCLD) bronchoalveolar lavage (BAL) samples (related to Figure 1). **(A-C)** Uniform manifold approximation and projection (UMAP) embedding of 55,776 PCLD BAL single-cell transcriptomes color coded by **(A)** cell type, **(B)** donor and **(C)** radiological phenotype. **(D)** Heatmap of up to the top 50 differentially expressed genes (Wilcoxon test, FDR<0.05) for each cell type and across subjects, colored by z-scores of log-normalized mRNA counts.

Supplementary Figure 2

A

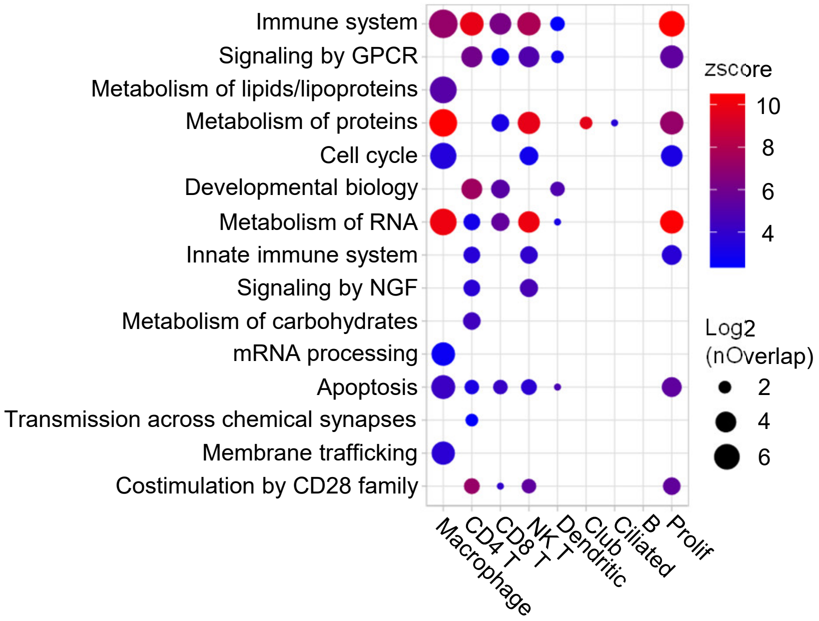

B

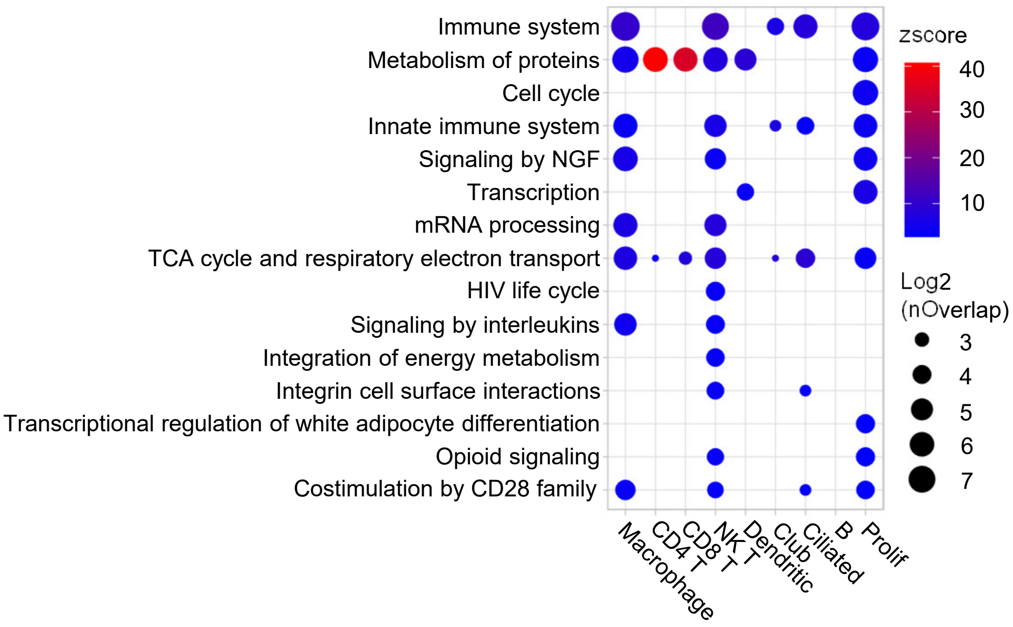

Biological pathways enriched within cell type-specific differentially expressed genes in each post-COVID-19 lung disease (PCLD) phenotype (related to Figure 1). Enrichment of Reactome pathways for cell type-specific genes expressed at significantly higher levels in **(A)** inflammatory PCLD and **(B)** fibrotic PCLD, identified by Wilcoxon test (FDR<0.05). Dot size represents the number of genes overlapping each biological pathway and colors reflect z-scores as an indicator of statistical significance.

Supplementary Figure 3

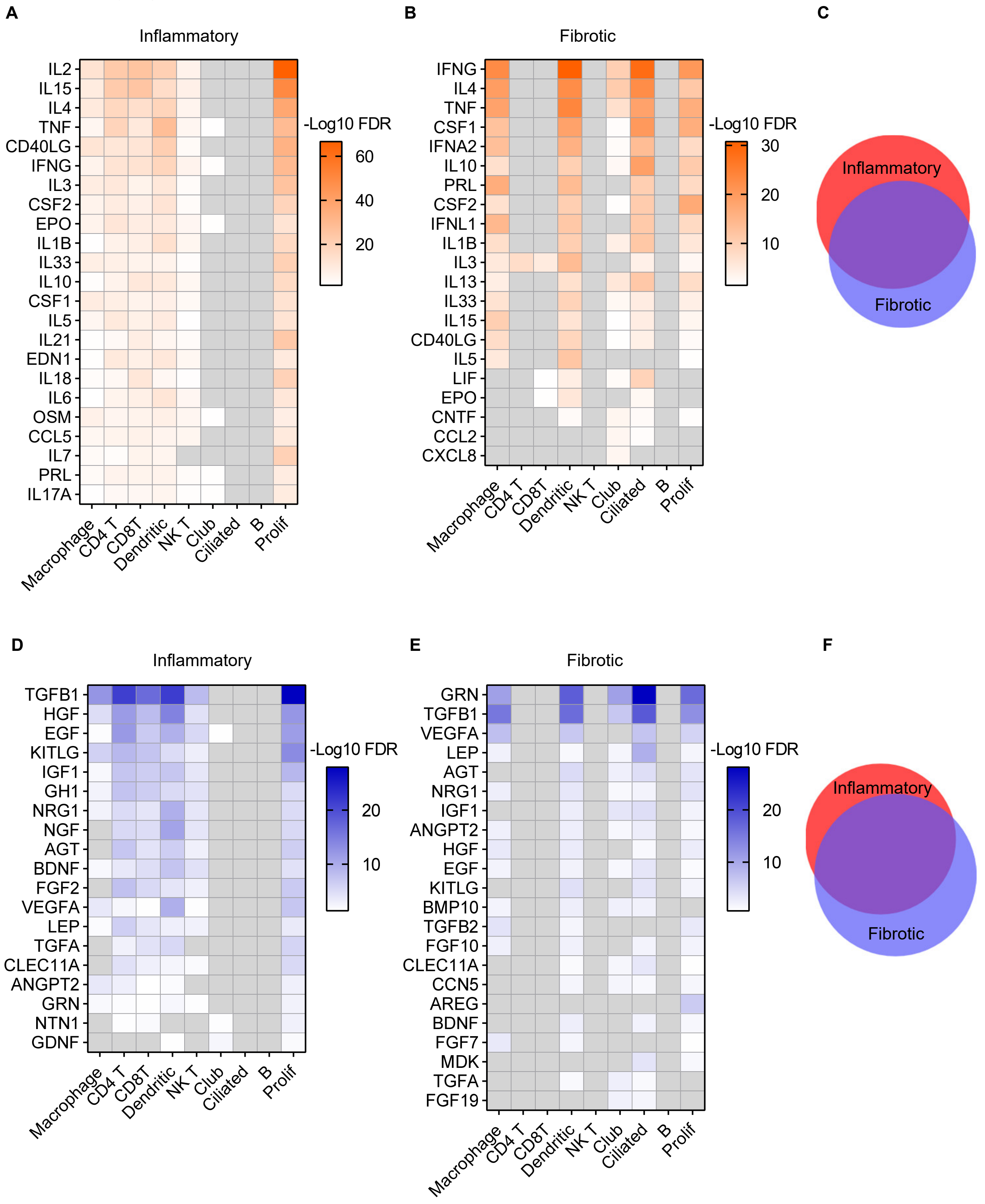

Predicted cytokine and growth factor upstream regulators of cell type-specific differential gene expression in post-COVID-19 lung disease (PCLD) (related to Figure 1). Heatmaps display integrated lists of the top 10 most statistically significant cytokines (orange) and growth factors (blue) across all cell types, predicted to regulate cell type-specific differentially expressed genes expressed more highly in **(A,D)** inflammatory PCLD and **(B,E)** fibrotic PCLD. Colors indicate statistical significance, represented by -log10FDR values. Grey heatmap cells indicate instances where molecules were not predicted to be upstream regulators of differential gene expression in that particular cell type. Area-proportional Venn diagrams represent the overlap of **(C)** cytokines and **(F)** growth factors predicted to regulate cell type-specific differential gene expression in inflammatory and fibrotic PCLD.

Supplementary Figure 4

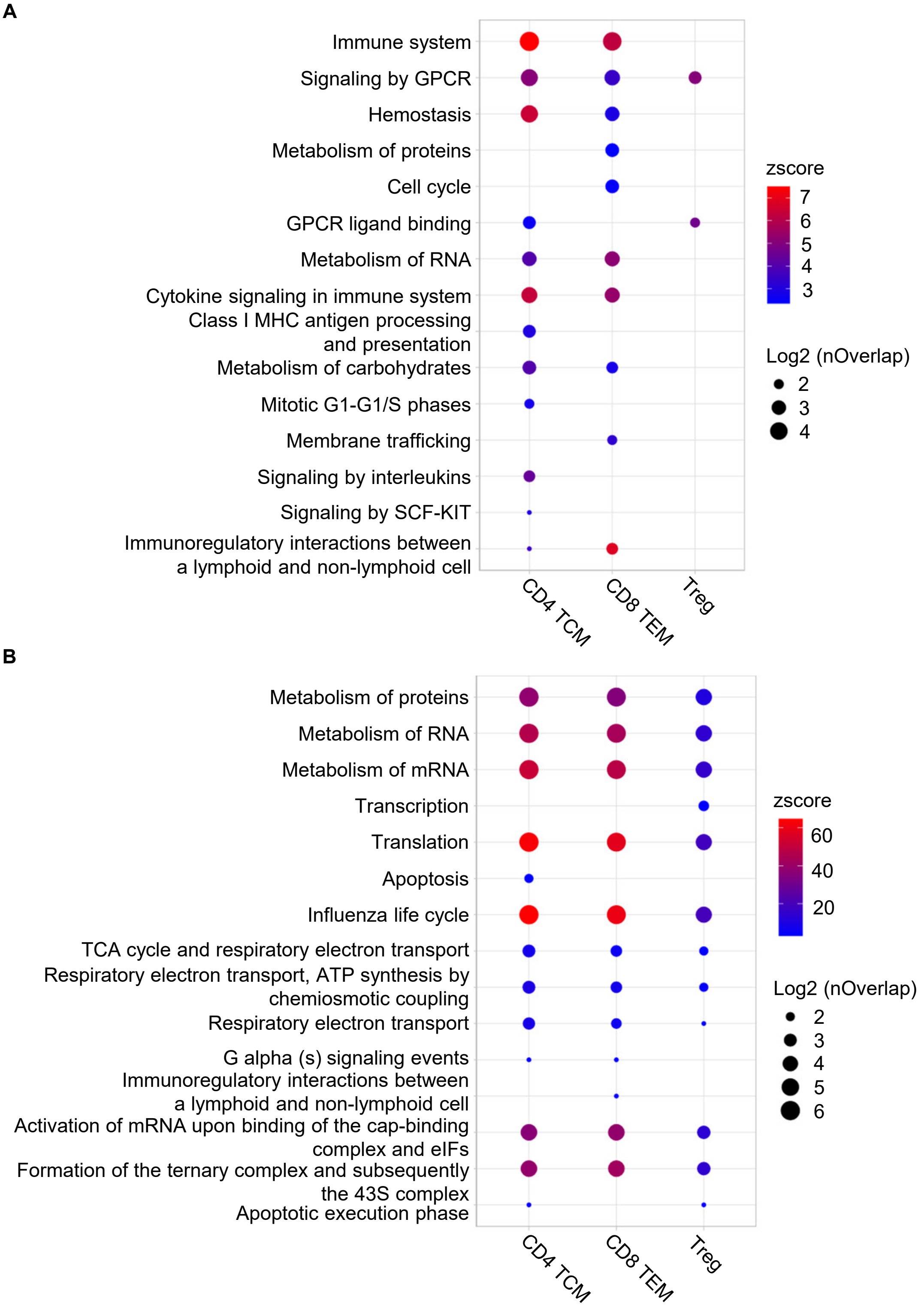

Enriched biological pathways among bronchoalveolar T cell subset-specific differentially expressed genes in post-COVID-19 lung disease (PCLD) (related to Figure 2). Enrichment of Reactome pathways for genes expressed at significantly higher levels in T cell subsets in **(A)** inflammatory PCLD and **(B)** fibrotic PCLD, identified by Wilcoxon test (FDR<0.05). Dot size represents the number of genes overlapping each biological pathway and colors represent statistical significance defined by z-score.

Supplementary Figure 5

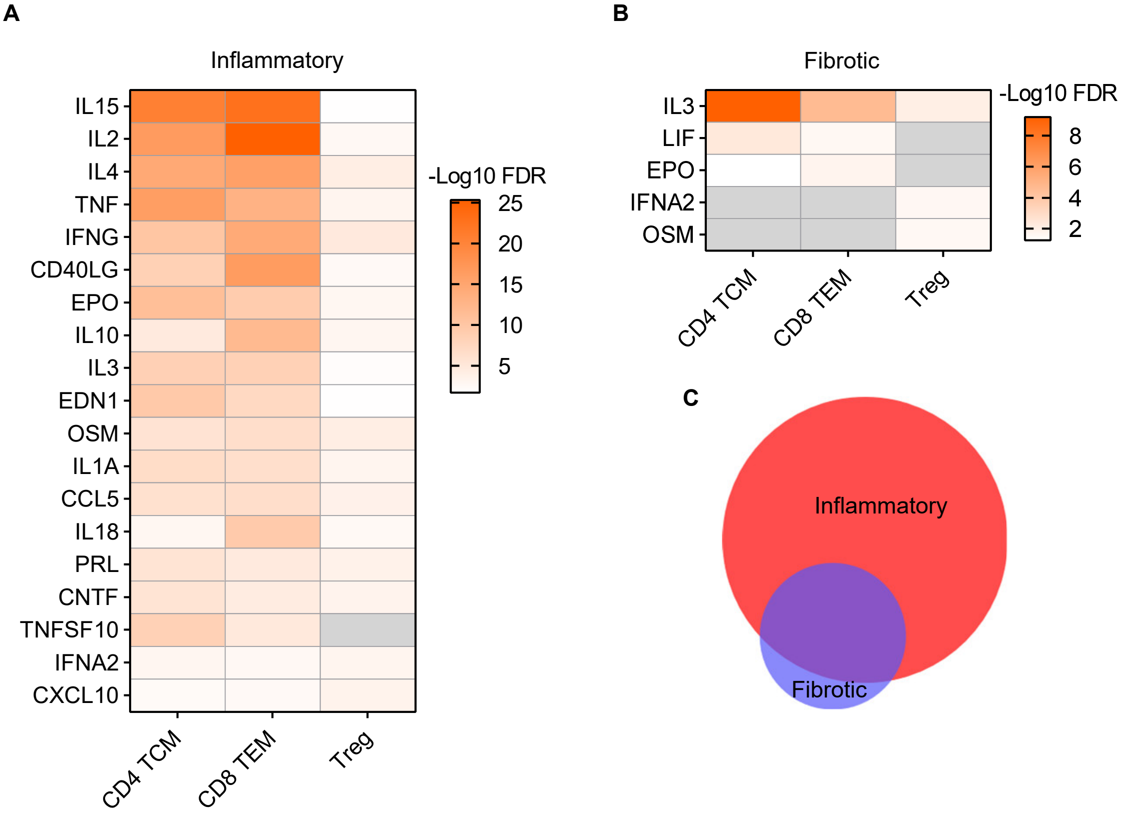

Predicted upstream regulation of T cell subset-specific differential gene expression in inflammatory and fibrotic post-COVID-19 lung disease (PCLD) (related to Figure 2). Heatmap visualisation of integrated lists of the top 10 most statistically enriched cytokines predicted to regulate differentially expressed genes specific to each of the three major T cell subsets expressed at higher levels in **(A)** inflammatory PCLD and **(B)** fibrotic PCLD. Colors indicate statistical significance, represented by -log10FDR values. Grey heatmap cells represent molecules not predicted to be upstream regulators of differential gene expression in a particular T cell subset. **(C)** Area-proportional Venn diagram representing the overlap of cytokines predicted to regulate T cell subset-specific differential gene expression in inflammatory and fibrotic PCLD.

Supplementary Figure 6

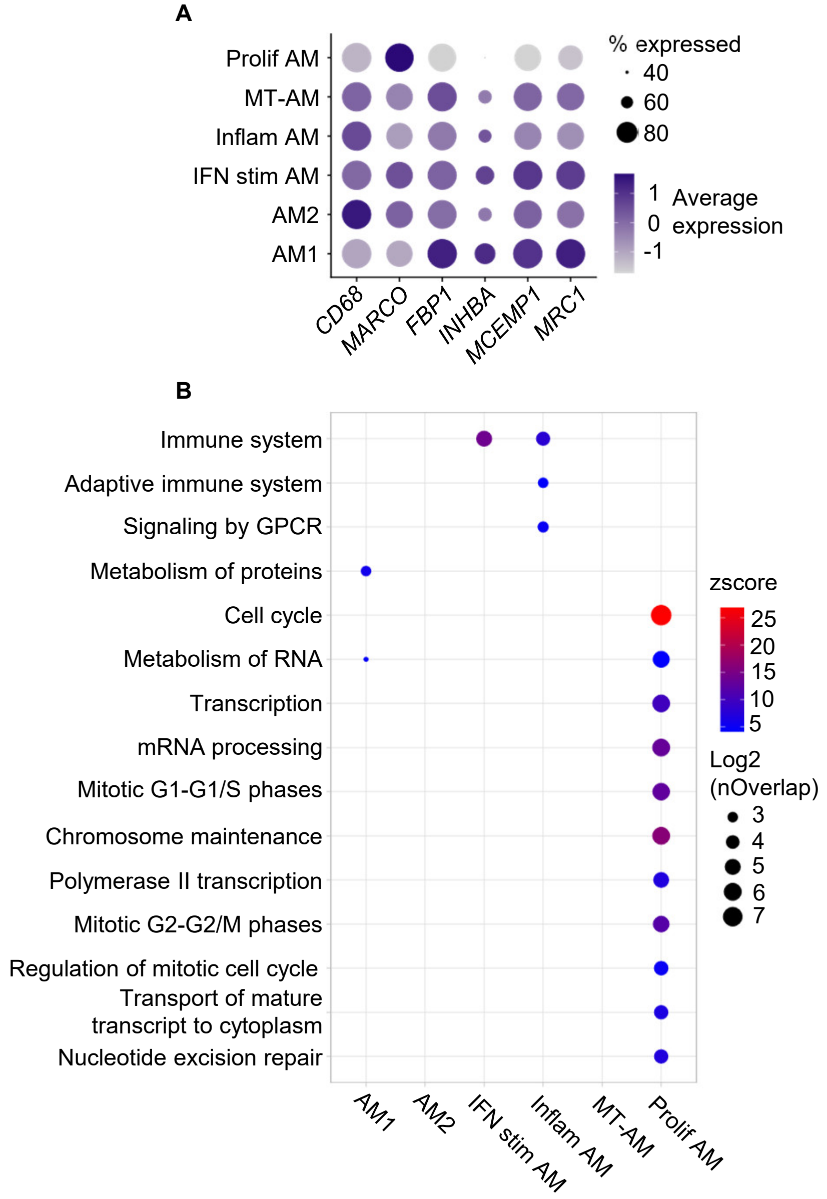

Macrophage marker gene expression and enriched biological pathways in post-COVID-19 lung disease (PCLD) myeloid populations (related to Figure 3). **(A)** Dot plot displays expression levels of independently established macrophage marker genes in airspace macrophage and monocyte subsets identified in PCLD. Dot size indicates the percentage of cells expressing the gene in each population, color represents average expression of scaled, log-normalized mRNA counts. **(B)** Enrichment of Reactome pathways for differentially expressed genes expressed more highly in each myeloid subset, identified by Wilcoxon test (FDR<0.05). Dot size represents the number of genes overlapping each biological pathway and colors represent statistical significance measured by z-score.

Supplementary Figure 7

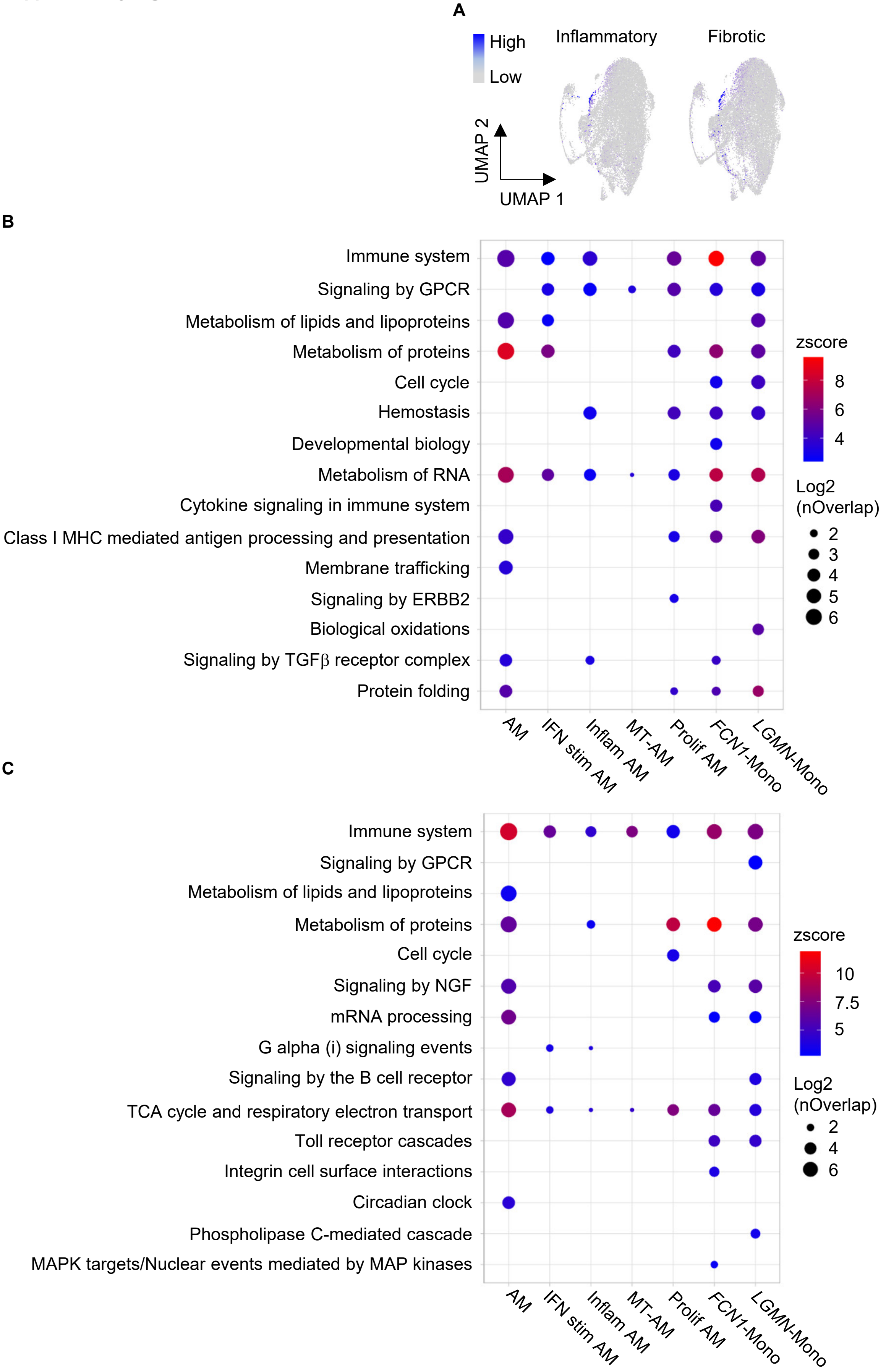

Biological pathways enriched among myeloid subset-specific differentially expressed genes in each post-COVID-19 lung disease (PCLD) phenotype (related to Figure 3). **(A)** Expression of a profibrotic macrophage gene signature derived from idiopathic pulmonary fibrosis, calculated on a single-cell level, colored by module score and projected on to the macrophage UMAP which is split by radiological phenotype. **(B,C)** Enrichment of Reactome pathways for genes expressed at significantly higher levels in each macrophage and monocyte subset in **(B)** inflammatory PCLD and **(C)** fibrotic PCLD, identified by Wilcoxon test (FDR<0.05). Dot size represents the number of genes overlapping each biological pathway and colors reflect z-scores as an indicator of statistical significance.

Supplementary Figure 8

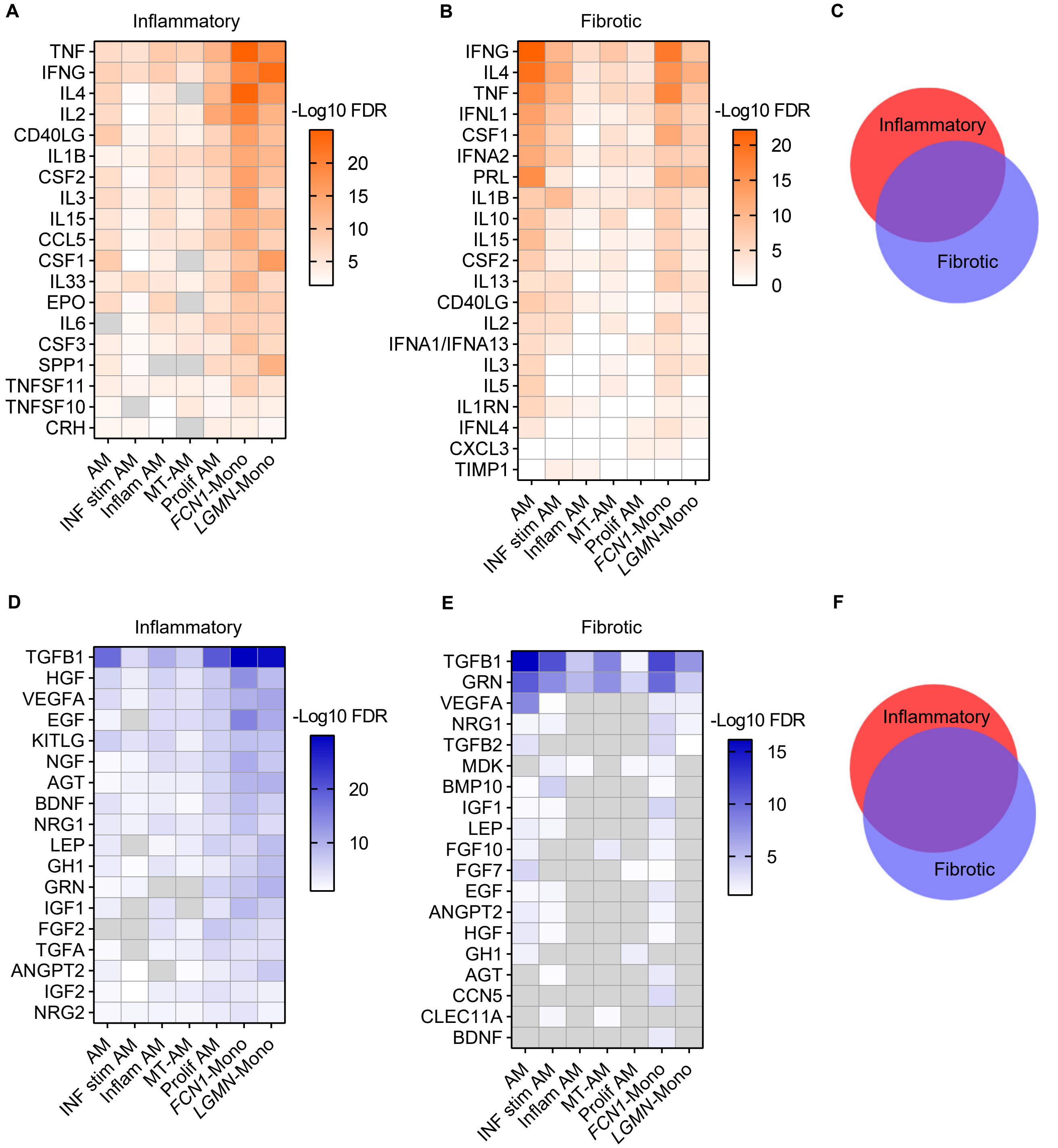

Predicted upstream regulation of differential gene expression in macrophage and monocyte subsets in inflammatory and fibrotic post-COVID-19 lung disease (PCLD) (related to Figure 3). Heatmaps depict integrated lists of the top 10 most statistically enriched cytokines (orange) or growth factors (blue) predicted to regulate differentially expressed genes expressed at higher levels each myeloid population in **(A,D)** inflammatory PCLD and **(B,E)** fibrotic PCLD. Colors indicate statistical significance, defined by -log10FDR values. Molecules not predicted to be upstream regulators of differential gene expression in a particular subset are displayed as grey heatmap cells. Area-proportional Venn diagrams represent the overlap of **(C)** cytokines and **(F)** growth factors predicted to regulate myeloid subset-specific differential gene expression in inflammatory and fibrotic PCLD.
